# Genome‐Wide Association Study of Grain Manganese Content in Bread Wheat (*Triticum aestivum* L.) Under Four Environments

**DOI:** 10.1002/fsn3.70170

**Published:** 2025-04-11

**Authors:** Wanting Li, Qi Zhang, Jiahao Wang, Yanyan Gao, Hui Zhang, Lina Jiang, Li Wang, Dejun Han, Jianhui Ma

**Affiliations:** ^1^ College of Life Sciences Henan Normal University Xinxiang Henan China; ^2^ State Key Laboratory of Crop Stress Biology in Arid Areas Northwest A&F University Yangling Shanxi China

**Keywords:** genome‐wide association study, microelement, wheat

## Abstract

Microelements play important roles to maintain the normal metabolism of the human body, and deficiency of microelements always leads to a serious diseases. Manganese (Mn) is an essential microelement for the human body. However, the mechanism for regulating grain Manganese (Mn) content (GMnC) in wheat is rarely studied. In this study, we determined the GMnC of 477 wheat accessions in four environments. The changes in GMnC in different released years and backgrounds were analyzed, which revealed that the trait of GMnC in wheat was not indirectly selected in the selective breeding process. The 660 K single nucleotide polymorphism microarray about these 477 wheat accessions was further used for a genome‐wide association study using the GMnC phenotype. Fifteen quantitative trait loci (QTLs) were identified on chromosomes 2A, 2B, 3B, 5A, 5B, 5D, 6A, 6D, 7A, and 7B for wheat GMnC, which were detected in more than two environments. Four important QTLs and some candidate genes were screened. This study will contribute to further understanding the mechanism in regulating GMnC in wheat.

## Graphical abstract

The GMnC of 477 wheat accessions under four environments was determined using ICP‐7000. The 477 wheat accessions were sequenced using a 660 K SNP microarray. GWAS was performed using the sequenced data and phenotypic data to identify the QTLs and candidate genes for GMnC in wheat.

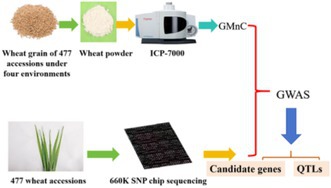

## 1 Introduction

Nutrient elements mainly refer to minerals that play substantive roles in the human body to maintain normal metabolism (Pujar et al. 2020). The essential elements for the human organism are categorized into macroelements and microelements. Among these, microelements participate in various biochemical reaction procedures in the human body. Deficiency of microelements always leads to a serious diseases, such as Anemia, slow growth, and impaired immune function (Yang et al. 2023). Nearly all the microelements are ingested into the human body through food. Therefore, it is of great significance to analyze and understand the molecular mechanism for regulating grain microelements content in important crops.

Bread wheat ( *Triticum aestivum* L.), one of the major food crops cultivated worldwide, provides essential nutrients such as proteins, minerals, and vitamins to more than 35% of the global population (Xu et al. 2023). Many researchers have focused on the content of microelements in wheat grain to improve wheat quality. Manganese (Mn) is an essential microelement for the human body. Mn deficiency can lead to symptoms such as fatigue, joint pain, decreased bone density, skin issues, and impaired immune system function (Rondanelli et al. 2021), whereas excessive Mn intake can induce neurotoxicity in matrix tissues (Gorell et al. 1997). The maximum daily intake of Mn should not exceed 11 mg per day (Mitchell et al. 2021). Therefore, the purpose of regulating grain Mn content (GMnC) is different from the improvement of iron or zinc content in wheat grain (Ma et al. 2023; Ma et al. 2024). The GMnC in wheat should be controlled within a certain range. Therefore, investigating the variation and genetic basis of GMnC in wheat is critically important.

Previous studies had been carried out to study the mechanism for transporting Mn into grains in different plants. However, only a gene regulating GMnC, *TaCNR5*, was functionally identified in wheat (Qiao et al. 2019). However, GMnC in wheat is a typical quantitative trait, and more genes deserve to be investigated. Genome‐wide association analysis (GWAS) is an important approach to screen key loci for quantitative traits, which would lay a foundation for further studies. A series of studies have been carried out to analyze key loci for microelements in wheat grains, such as Fe, Zn, Cu, Ca, and others, and many candidate genes for these traits were screened. For example, Velu et al. obtained 39 SNP markers for grain Zn content in wheat using GWAS, and some candidate genes, including zinc finger motifs of transcription factors and metal ion binding genes, were screened (Velu et al. 2018). In order to analyze the genetic basis of Ca accumulation in wheat grains, Shi et al. conducted GWAS on grain Cd content in wheat and found that the hot spot of genetic locus existed at the end of chromosome 4A. The candidate gene TraesCS4A02G428900 was highly expressed in wheat grains, which may be involved in Ca accumulation (Shi et al. 2022). Liu et al. identified 23 loci for grain Cu content on 15 chromosomes and 16 loci affecting grain Zn content on 11 chromosomes using 246 wheat accessions (Liu, Huang, et al. 2021; Liu, Chen, et al. 2021). Cu et al. used GWAS to analyze the QTLs for the contents of Zn, Fe, Cu, and P in wheat grains (Cu et al. 2020). From these researches, we found that many studies had been performed for the important microelements, such as Zn, Fe, Cu, and Ca. However, only six studies have been carried out for GMnC using GWAS in wheat (Morgounov et al. 2022; Wang et al. 2021; Cu et al. 2020; Hao et al. 2024; El‐Soda and Aljabri 2022; Bhatta et al. 2018). Compared with other microelements, the related research is obviously insufficient, and more work needs to be performed.

In this study, the GMnC of 477 wheat accessions planted in four environments was determined, and the change rule of GMnC was analyzed. The 660 K SNP chip was further applied to conduct GWAS, so as to find the QTLs for GMnC. It would provide important information for further understanding the genetic basis of GMnC in wheat.

## 2 Materials and Methods

### 2.1 Plant Materials and Cultivation

In this study, 477 wheat accessions were used to construct a wheat panel based on the wheat core germplasm resources, including 55 landrace, 69 exotic cultivars, 32 unknown, and 321 domestic cultivars. These wheat accessions were planted in four locations: Luoyang, Suqian, Nanyang, and Yangling; the field was managed according to local management practices as our previous description (Ma et al. 2023, 2024).

### 2.2 Determination of GMnC

At the maturity stage, three spikes were sampled as a biological replicate, and three biological replicates were obtained for each wheat accession across four environments, resulting in a total of 5724 samples. Each sample was washed with distilled water and ultrapure water, dried in an oven at 80°C, and ground into powder using a stainless steel grinder (MM400, Retsch, Haan, Germany). About 0.2 g of dried wheat powder was digested in a nitric acid and hydrogen peroxide mixture using a microwave digestion system (CEM MARS6 CLASSIC), then the finally digested water was fixed to 50 mL using ultrapure water. The Mn standard reserve solution from the National Standard Material Research Centre was obtained, and a standard curve was prepared for each round of determinations. The GMnC in each sample was determined using an inductively coupled plasma system (ICP‐7000, Thermo Scientifc).

### 2.3 Phenotyping, Genotyping, Population Structure, and Linkage Disequilibrium (LD) Analysis

The GMnC of wheat in four environments was used as the phenotypic data for the calculation of heritability (*H* ^2^) as our previously described (Ma et al. 2023). The 477 wheat accessions were genotyped using the Affymetrix wheat 660 K SNP array. The polyploid Affymetrix Genotyping Console (GTC) software was used to analyze the specific information of SNPs, including genotype identification, allele clustering, and SNP marking with Hardy–Weinberg value > 0.01. The probe was mapped to the IWGSC RefSeq v1.0 wheat reference genome using BLASTn (Basic Local Alignment Search Tool) 2.2.29+.

Model‐based population structure analysis was used to get the subgroups. Using STRUCTURE v2.3.4 software, population structure was assessed using unlinked markers (*r* ^2^ = 0) (Pritchard et al. 2000). The value of *K* is set from 2 to 20, indicating the number of subgroups. Five independent runs are performed for each *K* value, with an aging length of 20,000, and the number of iterations set to 10,000. The most likely subgroup number was determined using the Δ*K* method, based on the rate of change between *K* values (Earl and VonHoldt 2012). The average attenuation of genome A, B, and D was analyzed by PLINK software.

### 2.4 GWAS For Wheat GMnC

Univariate linear mixed model in GEMMA software was used to conduct GWAS. The recommended threshold for the *p* value (*p* = 1/Ne) was calculated using Bonferroni correction, where Ne represents the effective number of independent SNPs calculated using genetic Type I error calculator software (Li et al. 2012). –log10(*p* value) ≥ 3.5 of FarmCPU model was used to determine the significance of the SNPs. The SNP markers of GWAS were visualized using a Manhattan map.

## 3 Results

### 3.1 Phenotypic Variations for GMnC in Wheat

**FIGURE 1:**
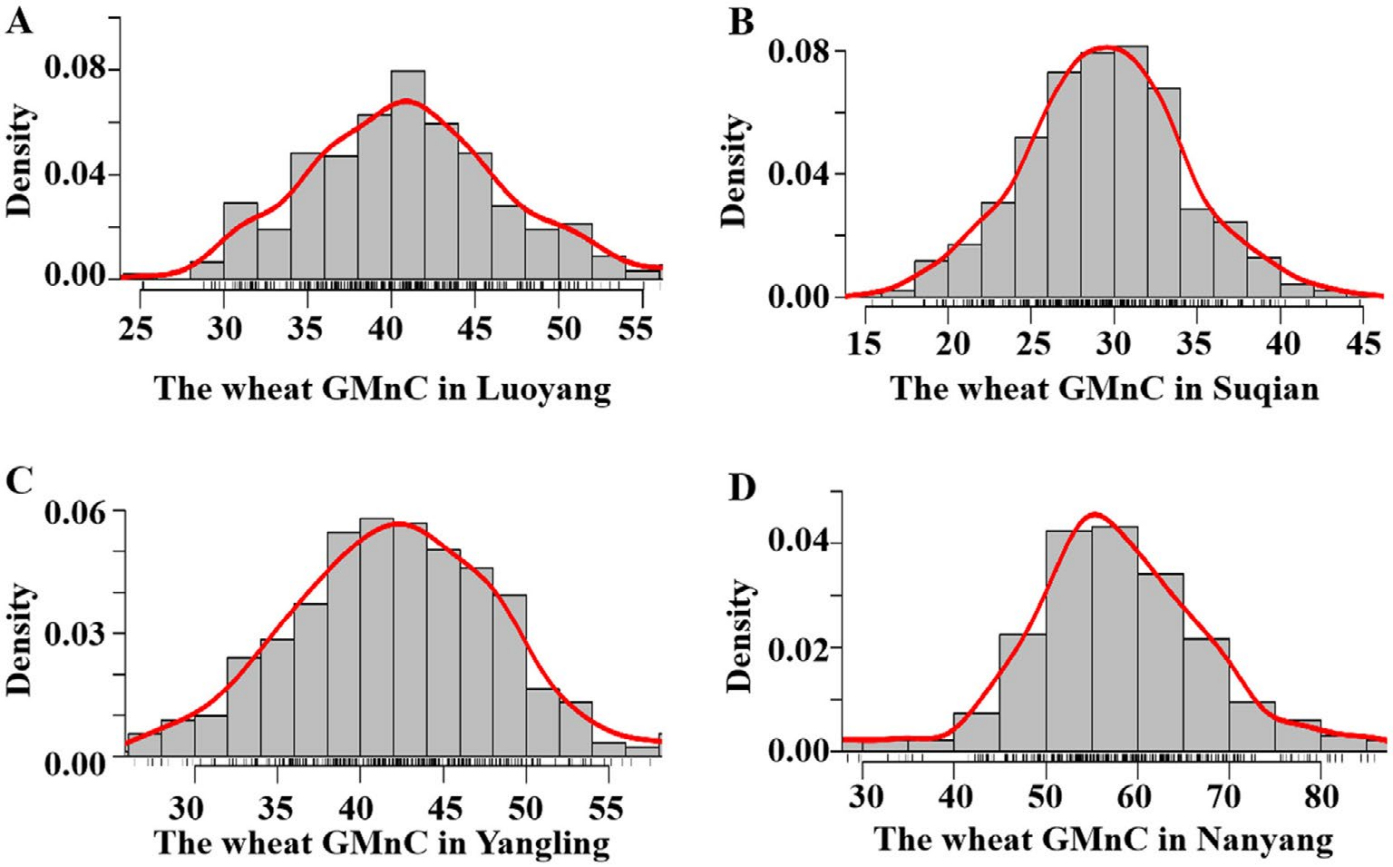
Wheat GMnC density in Luoyang (A), Suqian (B), Yangling (C), and Nanyang (D). The density cruve was represent by red curves, and the black short lines represent rug plot that represent the distribution of wheat GMnC.

The GMnC of 477 wheat accessions in four field environments was determined using ICP‐7000 (Table S1). The results exhibited that the highest mean value of GMnC was found in Nanyang (57.82 mg/kg) ranging from 24.29 to 95.88 mg/kg, followed by Yangling (44.50 mg/kg) and Luoyang (41.43 mg/kg). The lowest mean GMnC was observed in Suqian, with an average value of 30.10 mg/kg and a range of 15.41–82.80 mg/kg (Table 1). The overall variation coefficient ranged from 24.53% to 32.50%, and the standard deviation ranged from 7.50 to 14.46 mg/kg in the four environments (Table 1). Normal fitting revealed that the GMnC of 477 wheat accessions was normally distributed in different environments, which indicated a typical quantitative trait (Figure 1). The *H* ^2^ of GMnC was calculated to be 0.55, indicating that this trait was regulated by both genetic and environmental factors.

**TABLE 1:** The GMnC in four environments.

Environment	Min	Max	Mean	Variation coefficient (%)	Standard deviation	Skewness	Kurtosis
Yangling	15.73	112.25	44.50	32.50	14.46	2.31	8.20
Nanyang	24.29	95.88	57.82	24.53	14.18	0.06	1.21
Suqian	15.41	82.80	30.10	24.91	7.50	2.96	18.54
Luoyang	25.12	72.28	41.43	29.25	12.12	0.86	1.97

### 3.2 The Changes in Wheat GMnC in Different Released Years

**FIGURE 2:**
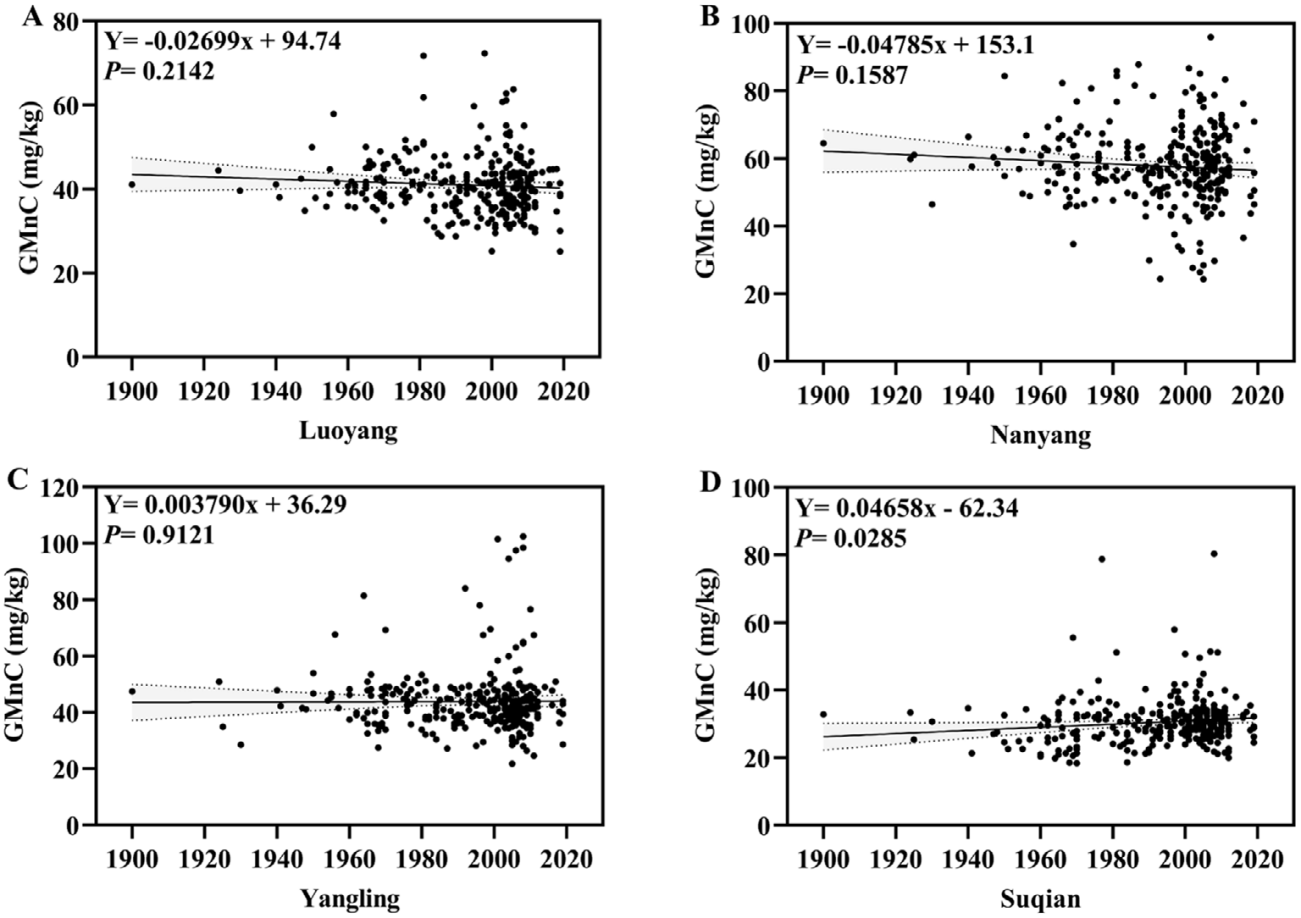
The relationship between wheat GMnC and released years in Luoyang (A), Nanyang (B), Yangling (C), and Suqian (D).

There were 477 wheat accessions in this study, and the released years of 306 wheat accessions of domestic wheat accessions were confirmed. To analyze the changes of GMnC with released years, a regression analysis was conducted in four environments (Figure 2). We found that there was no significant relationship between the GMnC and released years in Luoyang, Nanyang, and Yangling (Figure 2A–C). Only a poor relationship was found in Suqian (Figure 2D). These results indicated that the GMnC in wheat may not have been indirectly selected during the selection breeding process in the past decades.

### 3.3 The Genetic Diversity of SNPs in Three Subgenomes

The 660 K SNP array was used for conducting a GWAS using the phenotypes of GMnC of 477 wheat accessions. After the low‐quality SNPs (MAF < 0.05 and missing data > 0.1) were removed, 407,323 high‐quality SNPs unevenly distributed across 21 chromosomes were screened for further analysis. The number of effective SNP markers in the B subgenome was 39,631, which is the highest among the three subgenomes, followed by the A subgenome (36213) and the D subgenome (21386). The chromosome density of SNP markers showed that the number of SNP markers was the highest in 3B (8005.78) and the lowest in 4D (1715.98). The density distribution of SNP markers was not even, ranging from 5.83 markers per Mb for 4D chromosome to 66.53 markers per Mb for 3B chromosome. The marker densities of A, B, and D subgenomes were 33.01, 32.46, and 14.87, respectively. In addition, the polymorphism information content (PIC) values of 21 wheat chromosomes varied between 0.26 and 0.31 (Table 2).

**TABLE 2:** The SNP information for GWAS of 477 wheat accessions.

Chr	No. of markers	Effective number	Effective ratio	Suggestive *p* value	Markers (%)	Length (Mb)	Marker density	He	PIC
1A	28,930	5707.35	0.20	1.75E‐4	7.10	594.02	48.70	0.66	0.27
1B	20,683	4832.67	0.23	2.07E‐4	5.08	780.76	26.49	0.78	0.31
1D	10,636	3128.43	0.29	3.20E‐04	2.61	750.73	14.17	0.66	0.27
2A	28,906	6217.23	0.22	1.61E‐04	7.10	744.54	38.82	0.76	0.30
2B	29,000	6841.12	0.24	1.46E‐04	7.12	709.76	40.86	0.67	0.27
2D	10,557	3819.01	0.36	2.62E‐04	2.59	617.97	17.08	0.73	0.29
3A	19,478	4679.47	0.24	2.14E‐04	4.78	736.69	26.44	0.68	0.28
3B	45,860	8005.78	0.17	1.25E‐04	11.26	689.38	66.52	0.67	0.27
3D	7285	2705.20	0.37	3.70E‐04	1.79	801.25	9.09	0.70	0.28
4A	17,996	4332.16	0.24	2.31E‐04	4.42	830.70	21.66	0.71	0.28
4B	13,166	3072.23	0.23	3.25E‐04	3.23	673.47	19.55	0.72	0.29
4D	4157	1715.98	0.41	5.83E‐04	1.02	713.02	5.83	0.65	0.26
5A	22,698	5032.42	0.22	1.99E‐04	5.57	720.95	31.48	0.73	0.29
5B	34,064	6654.36	0.20	1.50E‐04	8.36	750.61	45.38	0.66	0.27
5D	8478	3306.79	0.39	3.02E‐04	2.08	495.44	17.11	0.68	0.27
6A	16,561	3880.80	0.23	2.58E‐04	4.07	651.81	25.41	0.69	0.28
6B	25,747	5647.72	0.22	1.77E‐04	6.32	615.48	41.83	0.78	0.31
6D	7507	2970.50	0.40	3.37E‐04	1.84	509.85	14.72	0.72	0.29
7A	28,342	6363.60	0.22	1.57E‐04	6.96	566.04	50.07	0.69	0.28
7B	17,140	4577.28	0.27	2.18E‐04	4.21	473.56	36.19	0.79	0.31
7D	10,132	3740.95	0.37	2.67E‐04	2.49	638.65	15.86	0.65	0.26
A genome	162,911	36213.03			40.00	4934.47	33.01	0.70	0.28
B genome	185,660	39631.16			45.58	5719.38	32.46	0.72	0.29
D genome	58,752	21386.86			14.42	3950.83	14.87	0.68	0.27
Total	407,323	97231.05				14064.68	28.96	0.70	0.28

### 3.4 The GMnC Analysis From Different Backgrounds

**FIGURE 3:**
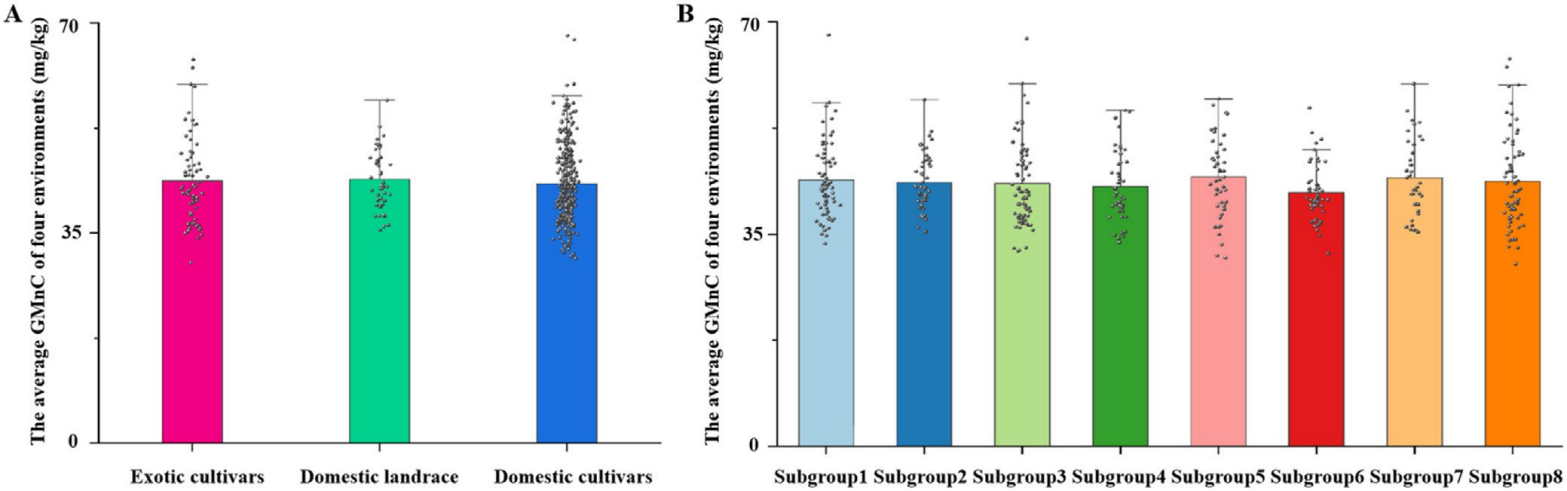
The average GMnC of four environments in different resource (A) and backgrounds (B). The pellets represent the specific GMnC value of the wheat accession belonging to the groups.

We analyzed the changes of wheat GMnC in different backgrounds. Based on the resources, the wheat GMnC in exotic cultivars, domestic landrace, and domestic cultivars was 43.71 ± 7.09 mg/kg, 42.96 ± 4.94 mg/kg, and 43.21 ± 4.88 mg/kg, respectively (Figure 3A), which showed no significant difference. The population structure analysis based on the Bayesian clustering method Δ*K* was further used, which resulted in eight subgroups, Subgroup 1–Subgroup 8 (Table S1). The wheat GMnC in eight subgroups was analyzed. The highest wheat GMnC was found in Subgroup 5, which mainly contained the wheat accessions released after 2000 in Hebei, Henan, Shanxi, and Shandong provinces, and the lowest GMnC was found in Subgroup 6, which was mainly composed of the wheat accessions in Sichuan, Jiangsu, and Hubei provinces. However, the wheat GMnC among the eight subgroups showed no significant difference (Figure 3B). These results proved the speculation by analyzing the wheat GMnC with released years, indicating that the wheat GMnC was not indirectly selected during the selection breeding process.

### 3.5 GWAS For GMnC in Wheat

**FIGURE 4:**
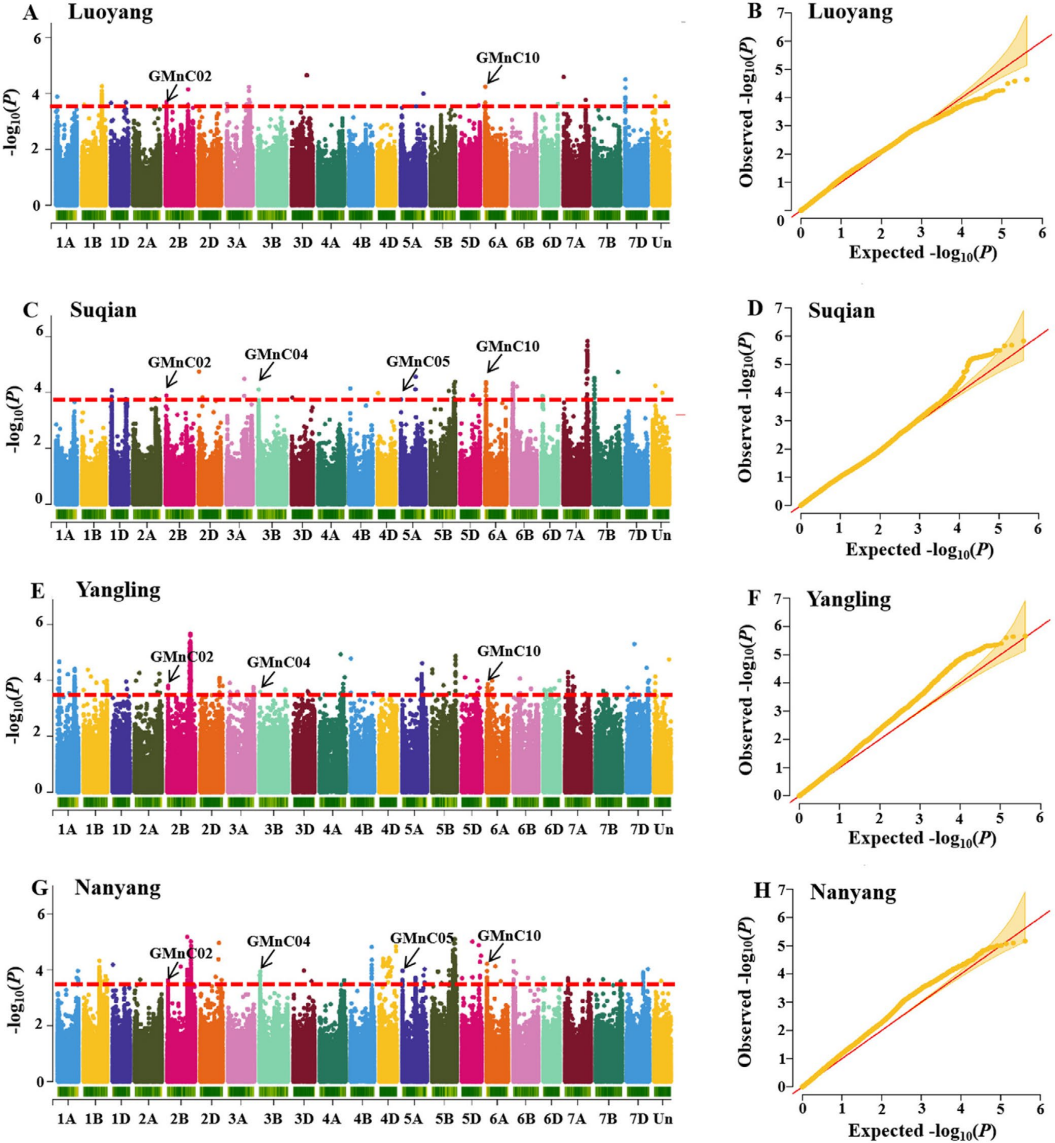
The Manhattan plots (A, C, E, G) and Q–Q plots (B, D, F, H) in Luoyang, Suqian, Yangling, and Nanyang. The significant SNPs with −log_10_ (*p* value) of > 3.5 are shown above the red lines. The four important QTLs were labeled in Manhattan plots.

In this study, the GMnC of 477 wheat accessions under four environments was used as the phenotypic data for GWAS using the FarmCPU model, and scatter plots were made using log (*p* value), with ≥ 3.5 as the significant SNPs (Figure 4A,C,E,G), and the Q–Q plots were also provided in Figure 4B,D,F,H. The QTLs with more than two environments were screened, and 15 QTLs were identified on chromosomes 2A, 2B, 3B, 5A, 5B, 5D, 6A, 6D, 7A, and 7B for wheat GMnC, which were named GMnC01 to GMnC15 (Table 3). Among them, 11 QTLs were identified in two environments, two QTLs were identified in three environments distributing on the chromosomes 2B and 3B, and two QTLs were identified in four environments distributing on the chromosomes 2B and 6A, which are highly likely to be the key loci for subsequent attention. To fully analyze the QTLs for wheat GMnC, we collected the previous research and got 126 SNP markers (MATs, Table S2). We integrated the 15 QTLs with 126 MATs and found that two QTLs, GMnC04 and GMnC05, overlapped with previous studies (Cu et al. 2020; Wang et al. 2021). Therefore, we finally got four important QTLs for GMnC, including two QTLs, GMnC02 and GMnC10, that were found in four environments and two QTLs, GMnC04 and GMnC05, overlapping with previous studies.

**TABLE 3:** The QTLs for GMnC in Luoyang (LY), Suqian (SQ), Yangling (YL), and Nanyang (NY).

ID	Chromosome	Marker	Favorable allele and its ratio	−Log_10_(*p*)	Environment
GMnC01	2A	AX‐110407685	T/C(0.89)	3.78	SQ/YL
GMnC02	2B	AX‐109465969	C/A(0.91)	3.88	LY/SQ/YL/NY
GMnC03	2B	AX‐109904766	A/T(0.92)	5.33	LY/YL/NY
GMnC04	3B	AX‐94599391	G/A(0.75)	4.10	SQ/YL/NY
GMnC05	5A	AX‐108906017	A/G(0.82)	3.96	SQ/NY
GMnC06	5A	AX‐108806718	C/A(0.85)	4.56	SQ/YL
GMnC07	5B	AX‐109537496	C/A(0.91)	3.97	YL/NY
GMnC08	5B	AX‐108730255	G/A(0.81)	4.87	SQ/YL
GMnC09	5D	AX‐109586097	C/T(0.93)	3.73	SQ/NY
GMnC10	6A	AX‐109815890	G/C(0.92)	4.24	LY/SQ/YL/NY
GMnC11	6A	AX‐110703144	G/T(0.93)	4.37	SQ/YL
GMnC12	6D	AX‐109579147	C/T(0.92)	3.87	SQ/YL
GMnC13	7A	AX‐111054494	T/C(0.76)	4.28	YL/NY
GMnC14	7A	AX‐109351037	A/G(0.80)	3.92	SQ/NY
GMnC15	7B	AX‐111542108	C/A(0.55)	3.66	YL/NY

Many genes had been functionally analyzed to participate in transporting metal iron in plants, such as HMA, PCS, and bHLH (Heiss et al. 2003; Meng et al. 2024; Wong and Cobbett 2009). In this study, there are 2032 genes in the 15 QTLs, and the annotation of these genes was provided in Table S3. Here, we found 2 PCSs, 1 HMA, and 12 bHLH among these 2032 genes. These may be the candidate genes for wheat GMnC for further research. The expression pattern of these candidate genes in different tissues was obtained from Table S4 using WheatOmics 1.0 (Ma et al. 2021). We found that the HMA showed high expression in the grain tissue, which may be an important candidate gene.

## 4 Discussion

Over the past few decades, global wheat production has risen from approximately 500 million tons to around 700 million tons, largely satisfying the demand for wheat consumption. Currently, the demand to improve wheat quality is quickly increasing with the improvement of living standards. Grain microelement content is an important indicator for wheat quality, and many researches were performed to improve this trait (Manickavelu et al. 2017). However, previous studies have predominantly concentrated on the improvement of Zn and Fe contents in wheat grains through genetic modification and cultivation measures (Ma et al. 2023, 2024), ignored other essential micronutrients. Mn is also a vital micronutrient for human health. More importantly, insufficient and excessive intake of Mn could cause several diseases (Avila et al. 2013). Therefore, understanding the GMnC in wheat is equally important.

In this study, we determined the GMnC of 477 wheat accessions under four environmental conditions. The mean wheat GMnC in four environments ranged from 30.11 to 67.89 mg/kg. Previous studies had analyzed the wheat GMnC in different environments. Cu et al. determined the wheat GMnC of 330 wheat lines cultured in Mexico during two successive crop seasons, and the wheat GMnC ranged from 38.7 to 74.1 mg/kg (Cu et al. 2020). Hao et al. collected 768 wheat accessions, which were cultured in two environments in China, to analyze the GMnC, and found that the GMnC ranged from 10.07 to 32.37 mg/kg (Hao et al. 2024). Liu et al. determined the GMnC of 161 lines from a cross between CN16 and D1 during two growing seasons, which were cultured in Chongzhou and Wenjiang of China, and found that the GMnC ranged from 16.28 to 53.85 mg/kg (Liu, Huang, et al. 2021). Bhatta et al. determined 123 synthetic hexaploid wheat cultured in Turkey during two growing seasons and found that the GMnC ranged from 20.3 to 69.8 mg/kg (Bhatta et al. 2018). Our result is in accordance with these researches. It was reported that the maximum recommended daily intake of Mn is 11 mg (Mitchell et al. 2021). With the increasing consumption of meat, eggs, and milk, the proportion of wheat flour in diets has decreased. According to the 8:1 ratio of the Mn content in bran to wheat flour, the maximum wheat flour consumption of the daily diet should not exceed 1 kg. Therefore, we recommend that the highest GMnC in wheat grain may be 99 mg/kg. In our results, we found that the GMnC for most wheat accessions in four environments was below 100 mg/kg, particularly those wheat accessions released after 2000. These results indicated that these wheat accessions for wheat production in China are reliable for the Mn nutrient element.

GWAS was widely used to identify the QTLs for grain microelement content in wheat, and many candidate genes were also identified (Cu et al. 2020; Hao et al. 2024; Liu, Huang, et al. 2021). In this study, we identified 15 QTLs for GMnC on 10 chromosomes (Table 3). Chromosome 6A was first identified to contain the QTLs for GMnC. Meanwhile, there are two QTLs that were identified in four environments, and two QTLs, GMnC04 and GMnC05, were found to overlap with previous research in chromosomes 3B and 5A by integrated analysis (Cu et al. 2020; Wang et al. 2021). Therefore, these QTLs should receive more attention in further studies. Importantly, we also identified 15 candidate genes in these QTLs according to the function annotation, which should receive more attention in further study.

In conclusion, we analyzed the changes in wheat GMnC with released years and different backgrounds and inferred that the trait of GMnC was not indirectly selected in the wheat breeding process. The GMnC in wheat was safe for people's consumption, relying on wheat as their staple food. Further GWAS and integrated analysis revealed four key QTLs for wheat GMnC, and some candidate genes were selected, which is of great significance for further analysis to understand the mechanism for transporting Mn to wheat grain.

## Author Contributions

**Wanting Li:** data curation (equal), investigation (equal), writing – original draft (equal). **Qi Zhang:** data curation (equal), investigation (equal), visualization (equal), writing – original draft (equal). **Jiahao Wang:** data curation (equal), investigation (equal), writing – original draft (equal). **Yanyan Gao:** data curation (equal), investigation (equal), writing – original draft (equal). **Hui Zhang:** data curation (equal), investigation (equal). **Lina Jiang:** data curation (equal), investigation (equal). **Li Wang:** conceptualization (equal), data curation (equal), investigation (equal), writing – original draft (equal). **Dejun Han:** conceptualization (equal), investigation (equal), writing – original draft (equal), writing – review and editing (equal). **Jianhui Ma:** conceptualization (equal), funding acquisition (equal), investigation (equal), writing – original draft (equal), writing – review and editing (equal).

## Conflicts of Interest

The authors declare no conflicts of interest.

## Supporting information


**Table S1.** Detailed information and GMnC of 477 wheat accessions.

**Table S2.** The previous identified SNP makers for wheat GMnC.

**Table S3.** The annotation of 2032 genes in 15 QTLs.

**Table S4.** The expression pattern of 15 candidate genes in different tissues using WheatOmics 1.0.

## Data Availability

The authors confirm that data generated or analyzed supporting the findings of this study are available within the article, and any further supporting material will be provided on request from the corresponding author.
